# Effect of Hydrolysable Tannins and Anthocyanins on Recurrent Urinary Tract Infections in Nephropathic Patients: Preliminary Data

**DOI:** 10.3390/nu13020591

**Published:** 2021-02-11

**Authors:** Annalisa Noce, Francesca Di Daniele, Margherita Campo, Manuela Di Lauro, Anna Pietroboni Zaitseva, Nicola Di Daniele, Giulia Marrone, Annalisa Romani

**Affiliations:** 1 UOC of Internal Medicine-Center of Hypertension and Nephrology Unit, Department of Systems Medicine, University of Rome Tor Vergata, Via Montpellier 1, 00133 Rome, Italy; francesca.didaniele@gmail.com (F.D.D.); dilauromanuela@gmail.com (M.D.L.); annapietroboni@icloud.com (A.P.Z.); didaniele@med.uniroma2.it (N.D.D.); giul.marr@gmail.com (G.M.); 2 PhD School of Applied Medical, Surgical Sciences, University of Rome Tor Vergata, Via Montpellier 1, 00133 Rome, Italy; 3 PHYTOLAB (Pharmaceutical, Cosmetic, Food Supplement, Technology and Analysis), DiSIA, University of Florence, Via Ugo Schiff 6, 50019 Sesto Fiorentino, Florence, Italy; margherita.campo@unifi.it

**Keywords:** hydrolysable tannins, anthocyanins, urinary tract infections, chronic kidney disease, quality of life, cranberry, Sweet Chestnut

## Abstract

Urinary tract infections (UTIs) are caused by uropathogenic microorganism colonization. UTIs often require an antibiotic therapy that can cause the selection of antibiotic-resistant bacterial strains. A natural bioactive compound may represent a valid therapeutic adjuvant approach, in combination with drug therapy. In this paper, we present a pilot study, based on the administration of an oral food supplement (OFS), containing chestnut tannins and anthocyanins, to nephropathic patients suffering from recurrent UTIs (16 treated patients with 1 cp/day and 10 untreated patients). We performed laboratory tests and quality of life and body composition assessments, at T0 (baseline) and T1 (after 6 weeks OFS assumption). The analysis of OFS was performed by HPLC-DAD-MS for its content in polyphenols and by in vitro tests for its antioxidative and anti-free radical activities. In each capsule, polyphenol content was 6.21 mg (4.57 mg hydrolysable tannins, 0.94 mg anthocyanosides, 0.51 mg proanthocyanidins, 0.18 mg quercetin derivatives). A significant reduction of erythrocyte sedimentation rate was observed only in male patients. Urinalysis showed a significant reduction of leukocytes in both genders, whereas urinary bacterial flora at T1 significantly decreased only in male subjects. Tannins seem to exert an antimicrobial action according to gender, useful to counteract the recurrence of UTIs.

## 1. Introduction

Urinary tract infections (UTIs) are a set of different clinical conditions due to the colonization of the urinary tract by uropathogenic microorganisms able to cause inflammatory and infective processes in the renal parenchyma or in excretory tract, as well. UTIs are the most frequent nephro-urological pathologies and represent the most common bacterial infections [1].

UTIs are a widespread global health problem and their prevalence is estimated at 0.7% worldwide [2]. UTIs occur more frequently in female subjects. In fact, a recent study observed that 40% of women present at least one episode of UTI during their lifetime and they have 30 times higher risk than men of developing UTIs [3].

The main UTI risk factors are age, female gender [4], sexual activity and the use of antibiotics [5]. Factors favoring the pathogenic invasion include anatomical abnormalities, medical devices such as urinary catheter, and favorable conditions related to the host (immunosuppression, diabetes mellitus, pregnancy). UTIs also represent a frequent condition in kidney transplant patients [6]. Furthermore, the onset of UTIs could have a genetic basis. Indeed, patients with a positive family history of UTI in first-degree relatives have an higher risk of developing them compared to the general population [7].

UTI can be caused by the invasion of various microorganisms, both Gram-negative and Gram-positive, and the most common pathogenic bacterium is *Escherichia coli* [8]. The diagnosis of UTI is made by combining symptoms with a positive urine culture [9]. In most patients, the threshold for bacteriuria is 1000 colony-forming units (CFU)/mL. However, in 20% of women with classic urinary symptoms, the urine culture can be negative, which mainly depends on the laboratory cut-off value [10].

Typical symptoms of UTIs can be systemic or local; the first include fever with chills and flank pain, and the second include dysuria, stranguria, pollakiuria, suprapubic pain and hematuria [11].

Recurrent UTIs are identified as two or more episodes of uncomplicated UTI of the lower urinary tract within the past 6 months, or 3 or more episodes over the past 12 months [12]. UTIs are a relatively frequent condition with a high impact on the quality of life and on healthcare costs, including visits, diagnostic tests and therapeutic prescriptions [13].

Recent studies have shown that recurrent UTIs are able to cause a worsening of the quality of life [14]. In fact, recurrent UTIs have a negative impact on daily habits, on sexual activity, on social and personal relationships, on the possibility of freely practicing sports, and on a decline in work productivity [15].

UTIs appear to be constantly growing in frequency, and this phenomenon is favored by the inappropriate use of antibiotics widely employed in both outpatient and hospital settings. This justifies the attention of research towards studies aimed to identifying new therapeutic strategies based on natural bioactive compounds, free from side effects such as nephrotoxicity or hepatotoxicity [16,17], and able to effectively counteract the recurrence of UTI.

Natural bioactive compounds exhibit well-known beneficial properties (such as antioxidants, anti-inflammatory and antimicrobial) which are mainly found in plant-based foods, such as fruit and vegetables [15,18,19,20]. Among these, the most studied are polyphenols, a wide group of substances that can be grouped into over 20 classes of organic compounds [21]. Recent studies suggest that long-term consumption of polyphenols, both in the form of fresh foods and oral food supplements, may have positive implications for human health. Specifically, several studies have demonstrated that polyphenols are able to reduce the incidence of chronic non-communicable diseases, such as cardiovascular diseases, obesity, diabetes mellitus, neurodegenerative diseases, chronic kidney disease (CKD) and some types of cancer [22,23,24,25].

Tannins belong to the class of secondary polyphenolic metabolites and they are found in a wide variety of foods, including cereals (such as sorghum, millet and barley) and legumes, but also in wine, green tea and coffee [26]. Thanks to their antioxidant and antimicrobial properties, tannins can be suitable for several innovative uses in various sectors, such as foods, cosmetics, phytotherapics, nutraceuticals and agronomics products [27].

Of clinical relevance are Chestnut tannins. In fact, several studies suggest that Chestnut tannins seem to have an important effect on human health, as they have known antioxidant, antitumor, antimicrobial, antifungal effects [28,29,30]. Tannins can also be involved in the reduction of triglycerides and total cholesterol levels and in the suppression of lipogenesis by insulin. Moreover, they present important astringent actions in the gastrointestinal tract [31]. In the literature, several studies are available concerning the antimicrobial activity of anthocyanins, proanthocyanidins and hydrolysable tannins from Cranberry and Sweet Chestnut. These studies report in vitro and in vivo actions towards bacteria and fungi such as *Escherichia coli*, *Klebsiella pneumoniae*, *Enterococcus faecalis*, *Pseudomonas aeruginosa*, *Candida* spp., which are among the main microorganisms responsible for UTIs. Moreover, both our and other research groups have previously tested these compounds in vitro as natural extracts, demonstrating that they can exert synergistic activities in combination with the traditional antibiotics or antifungals, or if administered as phytocomplexes [32,33,34,35,36,37,38,39]. For this reason, in the present study, an oral food supplement (OFS) containing extracts from Cranberry and Sweet Chestnut, was formulated and it was tested in vivo on patients with recurrent UTIs. The aim of our pilot study was to evaluate the anti-inflammatory, antimicrobial and antioxidant efficacy of hydrolysable Chestnut tannins and anthocyanins, administered as OFS, in a population of CKD patients affected by recurrent UTIs.

## 2. Materials and Methods

### 2.1. Oral Food Supplement

The studied OFS is referred to by the trade name “prosta-tan” and it is based on natural extracts rich in hydrolysable tannins obtained from Sweet Chestnut, furnished by Gruppo Mauro Saviola s.r.l. (Radicofani, Siena, Italy), Saviola Holding S.r.l. (Viadana, Mantova, Italy). Specifically, the OFS pharmaceutical form is a capsule containing a mixture of dry extracts from: *Castanea sativa* Mill. (22% p/p); *Serenoa repens* (W. Bartram) Small (20% p/p); *Vaccinium macrocarpon*, Ait. (11% p/p).

### 2.2. Chemicals

HPLC-grade solvents, formic acid (ACS reagent) and EGCG are from Sigma Aldrich Chemical Company Inc. (Milwaukee, WI, USA). Gallic acid and ellagic acid, cyanidin-3-*O*-glucoside chloride, quercetin and (±)-catechin hydrate, analytical grade, are from Sigma-Aldrich (St. Louis, MO, USA). HPLC-grade water was prepared via double-distillation and purification with a Labconco Water Pro PS polishing station (Labconco Corporation, Kansas City, MO, USA).

### 2.3. Extraction

Two extraction procedures at different pHs were optimized for anthocyanosidic and non-anthocyanosidic polyphenols. The powder present in one capsule was precisely weighed (416 ± 4 mg) and extracted in 4.0 m of a solution 70:30 EtOH:H_2_O acidified by HCOOH (pH 3.2 for non-anthocyanosidic polyphenols, pH 1.8 for anthocyanosides). The mixtures were kept under stirring at room temperature, protected from light, for 1h, then centrifuged at 5000 rpm for 5 min to separate the solid matrices from the extracts.

### 2.4. HPLC-DAD-MS Analysis

The analysis was performed on the extracts without dilution. The extracts were analyzed with a HP-1260 liquid chromatograph equipped with a DAD detector and a HP MSD API-electrospray (Agilent Technologies, Santa Clara, CA, USA) in negative and positive ionization mode. The chromatographic separation was performed by using a column Luna, C18 250 × 4.60 mm, 5 μm (Phenomenex, Torrance, CA, USA), operating at 26 °C. The eluents were H_2_O (pH 3.2 by HCOOH) and CH_3_CN. A four-step linear solvent gradient from 100% H_2_O up to 100% CH_3_CN was applied with a flow rate of 0.8 mL/min over a 55 min period, as previously described [40,41]. Mass spectrometer operating conditions were: gas temperature 350 °C, flow rate of 10.0 L/min, nebulizer pressure 30 psi, quadrupole temperature 30 °C and capillary voltage 3500 V. Fragmentor 120 eV. The identification was performed according to chromatographic, spectrometric and spectrophotometric data, by comparison with the specific standards available. Five-point calibration curves (r^2^ ≥ 0.999) were used, built with the specific standards. The correction of molecular weights was performed by multiplying each calibration result by the ratio between the molecular weight of the quantified compound and the molecular weight of the standard. Gallic acid and its derivatives were calibrated at 280 nm with gallic acid; ellagic acid and its derivatives were calibrated at 254 nm with ellagic acid; proanthocyanidins were calibrated at 280 nm with (±)-catechin hydrate, anthocyanosides were calibrated at 520 nm with cyanidin 3-*O*-glucoside; quercetin and its derivatives were calibrated at 350 nm with quercetin.

### 2.5. In Vitro Assays

Folin-Ciocalteu in vitro antioxidant capacity: Total phenols and polyphenols content was evaluated by spectrophotometric Folin-Ciocalteu assay, by measuring the absorbance at 725 nm of a sample solution containing Folin-Ciocalteu reagent, and 20% Na_2_CO_3_ after 40 min incubation. The five-point calibration curve was performed in gallic acid. The phenols content of each sample is reported as GAEs and correlated with the in vitro antioxidant activity [42,43].

In vitro assay with stable radical DPPH• (1,1-diphenyl-2-picrylhydrazyl): The antiradical activity was evaluated by stable radical DPPH• test, according to the previously reported procedure [44] with slight modifications. The extract was diluted and added 1:1 to an ethanolic solution of DPPH• (0.025 mg/mL).

The absorbance was measured at 517 nm with a DAD 8453 spectrophotometer (Agilent Technologies) at time 0 and every 2 min for 20 min. Antiradical activity % (AR%) was obtained through the relationship: [AR% = 100 × (A0 − A20)/A0]. A0 and A20 were the absorbance of DPPH• at 0 min and 20 min, respectively, after the addition of the diluted extract. The EC_50_ was the molar concentration in polyphenols of the solution that inhibits the DPPH• activity by 50%, determined by measuring the AR% for five different dilutions of the sample.

### 2.6. Patients

Twenty-six nephropathic patients affected by recurrent UTIs were recruited for the in vivo pilot study. Of these, sixteen patients (eight males and eight females) were treated with the OFS supplementation, as described below, and ten (five males and five females) represented the untreated subjects (control group). Each group were divided into two subgroups according to gender, homogeneous for age, body mass index (BMI) and CKD stage [45].

The inclusion criteria were age over 18 years, both sexes, signature and acceptance of informed consent, history of recurrent UTIs. The exclusion criteria were neoplastic subjects, patients with HIV positive infection, patients with liver disease and chronic viral hepatitis, patients with inflammatory and/or infectious pathologies in the acute phase, malnutrition (BMI < 18.5 kg/m^2^); pregnancy and end stage renal disease.

At the time of enrollment, the selected patients with recurrent UTI history allhad a negative urine culture but increased microbial flora and leukocytes in the urine sediment examination, in the absence of urinary symptoms.

**Figure 1:**
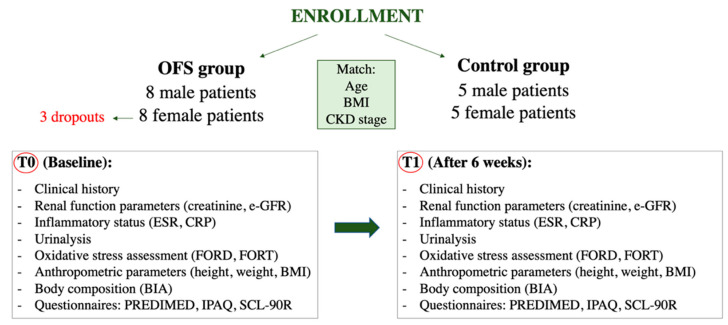
Flow-chart of in vivo pilot study. Abbreviations: BIA, bioelectrical impedance analysis; BMI, Body mass index; CKD, Chronic kidney disease; CRP, C reactive protein; e-GFR, Estimated-glomerula filtration rate; ESR, Erythrocyte sedimentation rate; FORD, Free oxygen radical defense; FORT, Free oxygen radical test; IPAQ, International physical activity questionnaire; OFS, Oral food supplement; PREDIMED, Prevención con Dieta Mediterránea; SCL-90R; Symptoms checklist-90 revised.

The patients of the OFS group were instructed to consume 1 capsule per day of OFS based on Chestnut tannins for six weeks. Blood and urinary parameters and the body composition assessments were monitored at two times during the study, at T0 (baseline) and at T1 (after six weeks), in both groups. Figure 1 shows the in vivo study flow-chart.

The study protocol complied with the declaration of Helsinki and was approved by the Ethical Committee of University Hospital Policlinico Tor Vergata (PTV) of Rome (project identification code 78/18 on 13 June 2018).

### 2.7. Laboratory Parameters

At baseline and after six weeks, we assessed the renal function through the evaluation of creatinine and estimated glomerular filtration rate (e-GFR). At the same time-points we evaluated the inflammatory status with C-reactive protein (CRP) and erythrocyte sedimentation rate (ESR). All patients underwent urinalysis to check UTIs signs. Furthermore, Free Oxygen Radical Test (FORT) and Free Oxygen Radical Defense (FORD) test were performed by CR4000, on capillary blood samples, to evaluate the oxidative stress [46] and the total antioxidant defense capacity [47], respectively.

A Dimension Vista 1500 (Siemens Healthcare Diagnostics, Milano, Italy) instrument was used to monitor all parameters. Standard enzymatic colorimetric techniques (Roche Modular P800, Roche Diagnostics, Indianapolis, IN, USA) were used to assess the lipid profile.

All other parameters were analyzed according to standard procedures of Clinical Biochemical Laboratories of University Hospital PTV of Rome.

### 2.8. Anthropometic and Body Composition Parameters

At the two time-points of the study (T0 and T1), an assessment of anthropometric parameters, such as height, weight and BMI, was performed. Body weight (kg) was measured to the nearest 0.01 kg with a balance scale (Seca 711, Hamburg, Germany), height (m) was measured with stadiometer to the nearest 0.1 cm (Seca 220, Hamburg, Germany). Standard methods were used to collect the anthropometric parameters [48]. BMI was calculated as body weight divided by height squared (kg/m^2^). Moreover, all enrolled patients underwent the evaluations of body composition by bioelectrical impedance analysis (BIA) using a BIA 101S instruments, Akern/RIL System-Florence. Resistance, reactance, impedance and phase angle at 50 KHz frequency were measured at T0 and T1. For the monitoring of hydration status, we evaluated total body water (TBW), intracellular water (ICW) and extracellular water (ECW) [49].

### 2.9. Questionnaires

To assess the possible biases induced by lifestyle changes, at baseline and at T1, we administered two questionnaires, the Prevención con Dieta Mediterránea (PREDIMED) questionnaire for the evaluation of adherence to the Mediterranean diet [50] and the International Physical Activity Questionnaire (IPAQ) for the evaluation of weekly physical activity [51], to all enrolled patients.

In addition, we administered a questionnaire for the evaluation of quality of life: the Symptoms Checklist-90 Revised (SCL-90R) [52]. SCL-90R assesses the presence and severity of psychological distress symptoms. Specifically, the questionnaire consists of 90 items and allows to detect nine different symptoms spheres: somatization, obsessive-compulsive disorder, interpersonal sensitivity, depression, anxiety, hostility, phobic anxiety, paranoid ideation and psychoticism [53]. The analyzed spheres of this study were somatization, anxiety and depression.

### 2.10. Statistical Analysis

All parametric variables are reported as means ± standard deviation, while non-parametric variables are reported as median (range minimum-maximum). We checked the normality of data for all continuous variables using the Kolmogorov-Smirnov test. The significance between T0 and T1 of parametric variables was tested with paired *t*-test, while the Wilcoxon test was used for the non-parametric variables. A *p*-value < 0.05 was considered statistically significant. The homogeneity of the subgroups was assessed using univariate ANOVA with a covariate for continuous parametric variables. Moreover, the short PREDIMED, IPAQ and SCL-90 data matrices were analyzed according to McNemar’s test [54]. Statistical analysis was performed with the Statistical Package for the Social Sciences Windows, version 15.0 (SPSS, Chicago, IL, USA). The graphic result visualization was obtained using GraphPad Prism (La Jolla, CA, USA).

## 3. Results

### 3.1. Supplement Characterization and In Vitro Study

**Figure 2:**
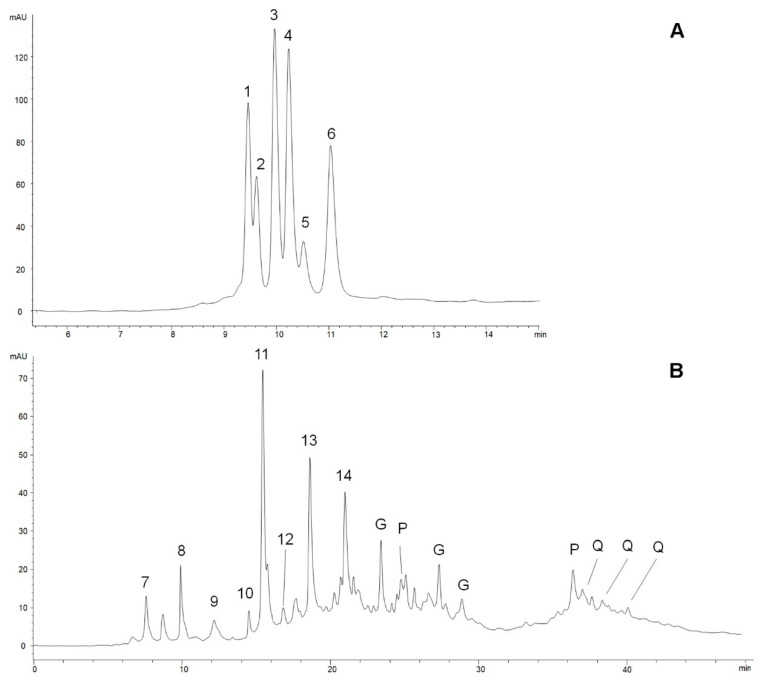
Chromatographic profiles of the OFS extracts (see Section 2). (**A**) pH 1.9, acquired at 520 nm: Anthocyanosidic compounds. 1. Cyanidin 3-*O*-galactoside; 2. Cyanidin 3-*O*-glucoside; 3. Cyanidin 3-*O*-arabinoside; 4. Peonidin 3-*O*-galactoside; 5. Peonidin 3-*O*-glucoside; 6. Peonidin 3-*O*-arabinoside. (**B**) pH 3.2, acquired at 280nm. Non-anthocyanosidic polyphenols. 7. Vescalin; 8. Castalin; 9. Pedunculagin I; 10. Monogalloyl glucose I; 11. Gallic acid; 12. Monogalloyl glucose II; 13. Vescalagin; 14. Castalagin; G. Gallic acid derivatives; P. Proanthocyanidins; Q. Quercetin derivatives.

The 1 h extraction procedure (see Section 2) was optimized and validated by comparing the quali-quantitative compositions of extracts prepared in the same conditions, but kept under stirring for 24 h, both for anthocyanosides and for the other polyphenols. Specifically, the OFS powder was extracted at pH 1.9 and pH 3.2 for 1 h and for 24 h. The HPLC-DAD-MS analyses (not reported here) showed a similar composition for the extracts at pH 3.2, whereas anthocyanosidic compounds extracted at pH 1.9 underwent a partial degradation with the longer time of extraction. Figure 2 A, B shows the chromatographic profiles of the two OFS extracts. The first one, acquired at 520 nm, is the profile of anthocyanosidic compounds extracted at pH 1.9, where six compounds were detected, identified and quantified (Table 1), the most abundant of which was cyanidin 3-*O*-arabinoside (0.435 ± 0.005 mg/g powder). Cyanidin was also found as its 3-*O*-galactoside and 3-*O*-glucoside (compounds 1–3 in Figure 2). Additionally, peonidin 3-*O*-galactoside, peonidin 3-*O*-glucoside and peonidin 3-*O*-arabinoside were present (compounds 4–6); peonidin 3-*O*-galactoside in the same amount as cyanidin 3-*O*-arabinoside. Total anthocyanosides were 1.89 ± 0.03 mg/g powder. These results are consistent with those previously reported in the literature for cranberry [55,56].

The second chromatographic profile, acquired at 280 nm, shows the presence of a large variety of non-anthocyanosidic polyphenols and two peaks of proanthocyanosidic compounds (“P” peaks). Total polyphenols extracted at pH 3.2 are 10.5 ± 0.2 mg/g powder: 9.1 ± 0.2 mg/g hydrolyzable tannins, 1.04 ± 0.03 mg/g proanthocyandins, 0.364 ± 0.008 mg/g quercetin derivatives. Gallic acid is the most represented polyphenol according to the number of moles, but the high molecular weights of the main hydrolysable tannins of Sweet Chestnut, vescalagin and castalagin, make them very abundant in weight, with vescalagin being the highest one (1.57 ± 0.02 mg/g powder) [40,41]. Quercetin derivatives were present in relatively low amounts, and come from the leaf component in Sweet Chestnut extract. The other hydrolysable tannins, complex gallic and ellagic acid esthers with glucose molecules, are typical compounds of Sweet Chestnut wood extracts, once generally indicated as “tannic acid”. Proanthocyanidins are typical of cranberry extracts, whereas fatty acids from Serenoa repens, its bioactive compounds, were not detectable and in any case cannot be efficiently extracted using the described procedures. Therefore, in one capsule containing 500 mg of powder, the total polyphenol content is 6.21 mg (4.57 mg hydrolysable tannins, 0.94 mg anthocyanosides, 0.51 mg proanthocyanidins, 0.18 mg quercetin derivatives, taking into account the absolute errors reported above).

Total antioxidant capacity and total polyphenols were evaluated by spectrophotometric assay with the Folin-Ciocalteu reagent, which allows for the determination of total phenols and polyphenols content through an electron-transfer (by H^+^ transfer) reaction between the sample under examination, in particular compounds with phenolic groups, and the Folin-Ciocalteu reagent. The results are calculated by using external calibration curves, usually in gallic acid, and expressed as mg/g GAE (Gallic Acid Equivalents). Thus, this test evaluates the total phenol compounds by determining the total antioxidant capacity in solution. The in vitro antioxidant activity showed a correlation between total phenols and minor polar compounds, as confirmed by previous studies carried out by comparing different electron transfer reaction assays (e.g., ferric reducing ability of plasma-FRAP, trolox equivalent antioxidant capacity-TEAC and oxygen radical absorbance capacity-ORAC) and in vitro assays on human low-density lipoproteins (LDL) [43,57]. The total phenol and polyphenol content in the examined OFS was 69.186 mg/g GAE.

The assay with DPPH• stable radical gave a measure of the antiradical activity of a sample, expressed as its EC_50_ (amount of sample inhibiting DPPH• activity to 50%). The EC_50_ of the OFS was calculated by measuring the antiradical activity of five different dilutions of the extract according to the procedure described in the “Materials and Methods” section, and calculating the molar concentration in polyphenols of the solution that inhibits the DPPH• activity by 50%. The measured EC_50_ was 0.251 ± 0.009 mg of OFS (3 µg polyphenols).

### 3.2. In Vivo Study

In the present pilot study, 16 patients with recurrent UTIs, 8 males (mean age 70 ± 2.5 years) and 8 females (mean age 61 ± 1.4 years), were enrolled as the OFS group, and 10 patients with recurrent UTIs, 5 males (mean age 69 ± 1.8 years) and 5 females (mean age 65 ± 2.0 years), were enrolled as the control group (untreated). The epidemiological parameters of the study populations and the evaluation of homogeneity based on gender in the two groups (OFS and control groups) are shown in Table 2.

Only five of the eight female treated patients completed the study protocol; three dropouts were recorded in female sex treated patients who complained of side effects in the gastrointestinal tract, such as epigastralgia, nausea and heartburn.

The laboratory parameters (T0 vs. T1) of the OFS group (males and females) are reported in Table 3. Assessment of renal function, monitored by creatinine and e-GRF, did not show statistically significant changes in either OFS subgroup. The evaluation of the inflammation indices showed a statistically significant reduction of ESR in male OFS patients (16.7 ± 2.2 mm/h vs. 11.3 ± 1.5 mm/h, *p* = 0.0062), while the reduction was not statistically significant in female OFS patients. In both genders, no significant reduction in CRP was observed.

Moreover, the urinalysis showed a reduction of leukocytes in the urinary sediment in both OFS subgroups (male: 43.5 (1–450) n/uL vs. 15 ± 5.7 n/uL, *p* = 0.0391; female: 28.5 (1–990) n/uL vs. 7 (1–91) n/uL, *p* = 0.0625). As regards the reduction of the urinary bacterial flora, a significant reduction was observed only in the male OFS subgroup (428 ± 143.4 n/uL vs. 34 (0–450) n/uL, *p* = 0.0156).

The laboratory parameters (T0 vs. T1) of the control group (males and females) are reported in Table 4. No statistically significant differences were shown between T1 and T0.

During the study period (6 weeks), we did not observe any UTI relapse in OFS population, while we detected three UTI relapses in the control group (two cases of *Escherichia.coli* and one case of *Enterococcus faecalis*).

The anthropometric parameters and the body composition assessment of OFS group were reported in Table 5, while those of control group were reported in Table 6. After six weeks of OFS treatment, no statistically significant differences were highlighted in either group.

At the end of the study, we observed a statistically significant decrease in oxidative stress monitored by FORT (261.4 ± 26.3 vs. 160 (160–250), *p* = 0.00391), and an increase in antioxidant defenses monitored by FORD (0.88 ± 0.1 vs. 1.43 ± 0.03, *p* = 0.0030) in male OFS patients, as reported in Table 7. We also observed a statistically significant increase in FORT (268 ± 46.2 vs. 312 ± 46.1, *p* = 0.0172) in female OFS patients. Oxidative parameters did not show statistically significant differences in the control group (Table 8).

We did not reveal any statistically significant differences in the patients’ lifestyle, monitored by PREDIMED questionnaire and IPAQ, as shown in Table 9.

**Figure 3:**
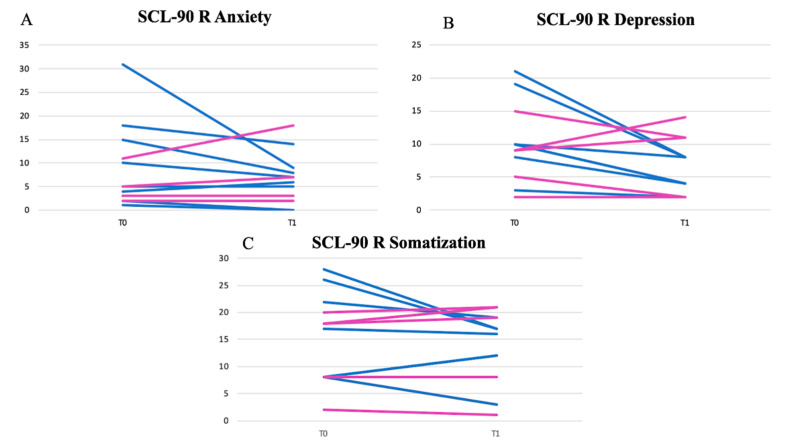
Results of anxiety (**A**), depression (**B**) and somatization (**C**) spheres of SCL-90 questionnaires of OFS group. Legend: blue lines for male patients; pink lines for female patients.

**Figure 4:**
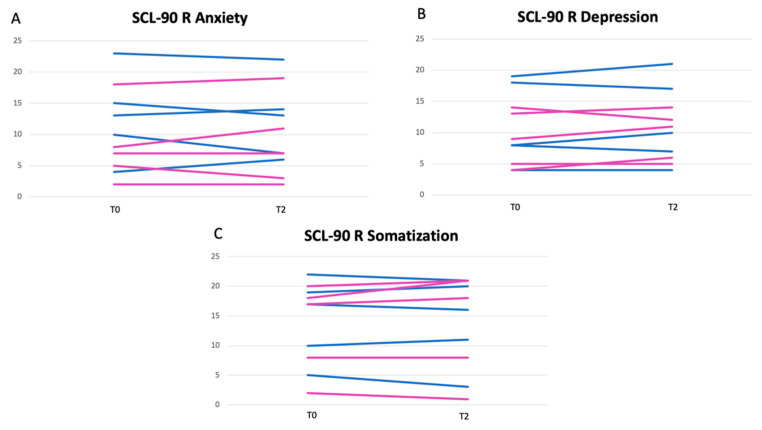
Results of anxiety (**A**), depression (**B**) and somatization (**C**) spheres of SCL-90 questionnaires of untreated group. Legend: blue lines for male patients; pink lines for female patients.

After six weeks, the psychological aspect was also assessed through the administration of the SCL-90R questionnaire. A statistically significant reduction in anxiety and depression spheres (and to a lesser extent in somatization) was observed in the male OFS subgroup. In female OGS subjects, this result was less evident. The results of SCL-90R of the OFS group are shown in Figure 3A–C, while the results of SCL-90R of the untreated group are shown in Figure 4A–C. The untreated group showed a slight worsening of the depressive and anxiety spheres, probably due to the chronic course of the disease.

## 4. Discussion

The hydrolysable tannins from Sweet Chestnut, in combination with the anthocyanosides and proanthocyanidins from Cranberry, seem to exert an antimicrobial action in a gender-dependent manner, useful for countering recurrent UTIs.

The monomers and aromatic acids derived from proanthocyanidins (the main phenolic metabolites of tannins) are found in the urine. Their absorption occurs at the gastrointestinal level, through the gut microbiota [26].

Ellagic acid and gallic acid are metabolites of proanthocyanidins and are found in the plasma, where they undergo conjugation processes with methyl, glucuronyl and sulphate groups and then they are excreted in the urine [58]. One hour after its oral intake, ellagic acid can be detected in the plasma.

A study by Seeram et al. [59] on 18 healthy subjects evaluated the presence of ellagic acid in the urine after pomegranate juice consumption. The authors highlighted that ellagic acid was not present in the plasma the previous day, while it was found both on the day and the day after the juice consumption in 24 h urine. Specifically, the gallic acid metabolites such as dimethylellagic acid glucuronide (DMEAG) and hydroxy-6H-benzopyran-6-one derivatives (urolithins) were detected in plasma in both free and conjugated form. Among the metabolites, the most important was urolithin A-glucuronide, which persisted in the urine for 48 h. This laid the basis for deducing that the daily intake of anthocyanidins and hydrolysable tannins for prolonged periods can exert an antimicrobial action in the urinary apparatus, counteracting relapses of UTIs.

In our pilot study, we administered Chestnut tannins and anthocyanins as OFS to evaluate their possible antimicrobial action on UTIs. This action was confirmed by the significant reduction in the urinary bacterial flora, in leukocyturia and in ESR, in male OFS patients. Cranberry standardized extracts and juices, rich in anthocyanidin/proanthocyanidin, demonstrated interesting effects in UTI prevention, as anthocyanidin/proanthocyanidin inhibit the adherence of P-fimbriated *Escherichia coli* to eukaryotic cells [32]. More recent studies have also demonstrated the efficacy of products containing Cranberry in inhibiting the in vitro growth of *Escherichia coli* strains, whereas the same products showed a lower activity compared to other pathogens like *Klebsiella pneumoniae*, *Enterococcus faecalis*, *Pseudomonas aeruginosa* and *Proteus mirabilis* [35,36,37,38,60].

Recent studies showed that Sweet Chestnut extracts and hydrolysable tannins present an antimicrobial activity against different pathogens. This finding suggests interesting and sustainable applications for food or feed safety and agronomics products [61,62,63,64,65,66]. In the biomedical field, tannic acid, tested in vitro both individually and in combination with fusidic acid on three strains of *Staphylococcus aureus* resistant to methycillin, showed a synergistic effect in preventing of additional adaptive mutations in the bacteria [39]. The chemical 1-methoxy-2,3-digalloylglucose, in mixture in the two anomeric forms, was tested both alone and in sub-inhibitory concentrations in combination with amphotericin B on *Candida albicans*, *Candida glabrata* and *Issatchenkia orientalis*, showing a strong synergistic activity [67].

A study on some terpenoids and on a wide variety of polyphenols, in particular hydrolysable tannins, selected among the representative molecules of natural extracts well known for their antibacterial properties, has confirmed their effectiveness against *Helicobacter pylori* of many of the tested compounds, in particular of hydrolysable tannins with MIC_50_s in plate between 6.25 and 50 µg/mL [33]. These results, together with those reported above, suggest the possibility of combining hydrolysable tannins from Sweet Chestnut and anthocyanosides/proanthocyanosides from Cranberry, for obtaining products with a wider spectrum of antimicrobial action and with possible synergistic effects. The above-reported in vitro and in vivo results, obtained with active compounds from Cranberry and Sweet Chestnut on microorganisms responsible for UTIs, by both our and other research groups, led us to the innovative formulation of the OFS object of the present pilot study.

In the male OFS subgroup in our study, a statistically significant improvement of the parameters related to oxidative stress (FORT and FORD) was also described, according to the high antioxidant activity and the low EC_50_ antiradical activity, measured using in vitro assays with Folin-Ciocalteu reactive and stable radical DPPH∙, respectively.

Such results can be ascribed to the presence of *Serenoa repens* present in the OFS in addition to Chestnut tannins and anthocyanosides. *Serenoa repens* (also commonly called saw palmetto) is a ripe berry of the North American dwarf-palm, traditionally used as treatment for the main male urogenital disturbances. Previous studies have highlighted that *Serenoa repens* has an antispastic, anti-edema, anti-proliferative and anti-androgenic effect [68]. Moreover, *Serenoa repens* extract, in particular its free fatty acid, such as lauric acid and linoleic acid, seems to exert an anti-inflammatory action through inhibition the cyclooxygenase activity, 5-lipoxygenase pathway and pro-inflammatory cytokines biosynthesis [59,60,61,62].

However, these promising effects did not seem to be visible in female treated patients. Furthermore, in these subgroups, the OFS seems to have had side effects in the gastrointestinal tract. The OFS tested, due to the high content of *Serenoa repens*, could have induced nausea, vomiting and other minor gastrointestinal symptoms in female subjects as a result of overdose with respect to their body weight [69]. The presence of this substance could explain the reason we observed cases of dropout in female treated subjects. Three female treated subjects, in fact, did not complete the study due to reported gastrointestinal disorders, leading us to hypothesize that the amount of *Serenoa repens* present in the OFS represented an overdose.

Furthermore, in male OFS patients, we observed a reduction in somatization, anxiety and depression state at the end of the study (after six weeks of OFS assumption). These results seem to be in line with the results obtained on the improvement of urinary symptoms in male subgroups. Gender differences have been reported for polyphenols and other bioactive compounds, related to their biotransformation, bioavailability, pharmacodynamics and pharmacokinetics. The metabolism of polyphenolic compounds takes place via gut microbiota and via endogenous enzymes such as cytochrome P450 mono-oxygenases in a gender-dependent manner. The specific targets of these bioactive compounds can be differently expressed in the different genders. Their renal excretion, which is the main excretion route, presents sex differences [70,71,72,73,74].

Thus, the gender is a variable that must be carefully considered. From this perspective, and according to the results of this pilot study, the research will continue with the experimentation of two different OFSs, specific for gender: the OFS (described above) for male patients and a newly formulated OFS, avoiding the use of *Serenoa repens* extract, for female patients.

## 5. Conclusions

The psychophysical distress induced by UTI recurrence, the possible negative effects related to repeated antibiotics treatment, and the possibility of antibiotic-resistance, have led to interest in finding a natural OFS designed to counteract recurrent UTIs and improve the quality of life of patients. The preliminary results of our pilot study demonstrate the possible therapeutic and preventive efficacy of a natural OFS based on polyphenols, specifically based on Sweet Chestnut tannins, in UTI recurrent patients. However, these results seem to be referable only to male patients. The significant side effects associated with the poor ability to reduce microbial flora in female patients raises the problem of finding a natural OFS able to counteract UTIs in female subjects. To confirm the results obtained for the parameters related to oxidative stress, inflammatory status and gender-dependent antimicrobial activity, a future study should be planned on a larger sample of patients selected by gender, also including a placebo group not treated with tannins. This study should take into account gender differences, formulating two OFS that differ with respect to the presence of *Serenoa repens* extract, which is not completely tolerated by the female population.

## Figures and Tables

**Table 1:** Polyphenol content in the tested OFS. Results in mg/g powder, with absolute errors.

Polyphenols	mg/g
Cyanidin 3-*O*-galactoside	0.347 ± 0.004
Cyanidin 3-*O*-glucoside	0.205 ± 0.003
Cyanidin 3-*O*-arabinoside	0.435 ± 0.005
Peonidin 3-*O*-galactoside	0.435 ± 0.006
Peonidin 3-*O*-glucoside	0.066 ± 0.002
Peonidin 3-*O*-arabinoside	0.397 ± 0.005
Vescalin	0.51 ± 0.01
Castalin	0.340 ± 0.009
Pedunculagin I	0.705 ± 0.008
Monogalloyl glucose I	0.198 ± 0.005
Gallic acid	1.34 ± 0.03
Monogalloyl glucose II	0.65 ± 0.02
Vescalagin	1.57 ± 0.02
Castalagin	1.15 ± 0.03
Gallic acid derivatives	2.68 ± 0.04
Proanthocyanidins	1.04 ± 0.03
Quercetin derivatives	0.364 ± 0.008
**Total polyphenols**	**12.4 ± 0.2**

**Table 2:** Epidemiological findings of study populations (OFS and control groups) and evaluation of the homogeneity divided according to gender.

	OFS Patients			Control Group		
	Males	Females	*p* (ANOVA Test)	Males	Females	*p* (ANOVA Test)
**N**	8	8		5	5	
Age (years)	70 ± 2.5 ^a^	61 ± 1.4 ^a^	ns	69 ± 1.8 ^a^	65 ± 2.0 ^a^	ns
Weight (kg)	74.2 ± 4.6 ^a^	73.9 ± 3.5 ^a^	ns	73.1 ± 3.9 ^a^	73.5 ± 3.4 ^a^	ns
BMI (kg/m^2^)	26.6 ± 1.8 ^a^	26.0 ± 1.7 ^a^	ns	26.1 ± 1.9 ^a^	25.8 ± 1.8 ^a^	ns

^a^ Data expressed as mean ± standard deviation; Abbreviations: ns = not significant. OFS = Oral food supplement.

**Table 3:** Laboratory parameters of two subgroups of OFS patients.

		Male Patients			Female Patients	
	T0	T1	T0 vs. T1	T0	T1	T0 vs. T1
Creatinine (mg/dL)	1.57 ± 0.8 ^a^	1.51 ± 0.9 ^a^	ns ^b^	1.1 ± 0.2 ^a^	0.8 ± 0.1 ^a^	ns ^b^
e-GFR (mL/min/1.73 m^2^)	43.0 ± 2.5 ^a^	46.55 (36 − 49) ^c^	ns ^d^	64.5 ± 10.0 ^a^	80.0 ± 12.0 ^a^	ns ^b^
CRP (mg/L)	1.0 (0.5 − 7.5) ^c^	0.9 (0.5–3.4) ^c^	ns ^d^	1.1 (0.3–10.1) ^c^	1.5 (1.1–8.4) ^c^	ns ^d^
ESR (mm/h)	16.7 ± 2.2 ^a^	11.3 ± 1.5 ^a^	0.0062 ^b^	27 ± 7.7 ^a^	28.2 ± 5.3 ^a^	ns ^b^
Urine pH	6 (5.5–7.5) ^c^	6.5 (5.5–7.5) ^c^	ns ^d^	6.3 ± 1.0 ^a^	5.6 ± 0.4 ^a^	ns ^b^
Urinary erythrocytes (n/uL)	10 (0–25) ^c^	7 (4–24) ^c^	ns ^d^	4.5 (1–49) ^c^	1 (1–7) ^c^	ns ^d^
Urinary leukocytes (n/uL)	43.5 (1–450) ^c^	15 ± 5.7 ^a^	0.0391 ^d^	28.5 (1–990) ^c^	7 (1–91) ^c^	0.0625 ^d^
Urinary bacterial flora (n/uL)	428 ± 143.4 ^a^	34 (0–450) ^c^	0.0156 ^d^	553 (36–22,807) ^c^	559 (16–61,990) ^c^	ns ^d^

^a^ Data expressed as mean ± standard deviation; ^b^ Applied test: *t*-test for paired data. ^c^ Data expressed as a median and the minimum-maximum range is shown in brackets; ^d^ Applied test: Wilcoxon test; Values of *p* ≤ 0.05 are considered statistically significant. Abbreviations: e-GFR, estimated glomerular filtration rate; TC, total-cholesterol; HDL-C, high-density lipoprotein cholesterol; LDL-C, low-density lipoprotein cholesterol; CRP, C-reactive protein; ESR, erythrocyte sedimentation rate; PTH, parathyroid hormone; ns, not significant.

**Table 4:** Laboratory parameters of control group divided in two subgroups according to gender.

		Male Patients			Female Patients	
	T0	T1	T0 vs. T1	T0	T1	T0 vs. T1
Creatinine (mg/dL)	1.60 ± 0.7 ^a^	1.59 ± 0.8 ^a^	ns ^b^	1.2 ± 0.1 ^a^	1.1 ± 0.2 ^a^	ns ^b^
e-GFR (mL/min/1.73 m^2^)	43.4 ± 2.7 ^a^	43.6 ± 2.8 ^a^	ns ^b^	47.4 ± 9.5 ^a^	52.6 ± 11.2 ^a^	ns ^b^
CRP (mg/L)	1.1 (0.6–6.8) ^c^	1.0 (0.4–3.2) ^c^	ns ^d^	1.2 (0.3–10.5) ^c^	1.3 (1.0–7.6) ^c^	ns ^d^
ESR (mm/h)	11.4 ± 1.9 ^a^	11.0 ± 1.6 ^a^	ns ^b^	15.7 ± 5.1 ^a^	14.0 ± 5.2 ^a^	ns ^b^
Urine pH	6.2 ± 1.1 ^a^	6.5 ± 1.2 ^a^	ns ^b^	6.2 ± 1.0 ^a^	5.9 ± 0.6 ^a^	ns ^b^
Urinary erythrocytes (n/uL)	9 (4–25) ^c^	7 (5–21) ^c^	ns ^d^	5 (1–30) ^c^	2 (1–9) ^c^	ns ^d^
Urinary leukocytes (n/uL)	33.5 (1–45) ^c^	36.0 ± 5.6 ^a^	ns ^d^	20.5 (1–70) ^c^	22.9 (1–50) ^c^	ns ^d^
Urinary bacterial flora (n/uL)	428 ± 143.4 ^a^	430 ± 140.4 ^a^	ns ^b^	543 (46–21,700) ^c^	552 (20–22,980) ^c^	ns ^d^

^a^ Data expressed as mean ± standard deviation; ^b^ Applied test: *t*-test for paired data. ^c^ Data expressed as a median and the minimum-maximum range is shown in brackets; ^d^ Applied test: Wilcoxon test; Values of *p* ≤ 0.05 are considered statistically significant. Abbreviations: e-GFR, estimated glomerular filtration rate; TC, total-cholesterol; HDL-C, high-density lipoprotein cholesterol; LDL-C, low-density lipoprotein cholesterol; CRP, C-reactive protein; ESR, erythrocyte sedimentation rate; PTH, parathyroid hormone; ns, not significant.

**Table 5:** Body composition assessment of two subgroups of OFS patients.

		Male Patients			Female Patients	
	T0	T1	T0 vs. T1	T0	T1	T0 vs. T1
Weight (kg)	74.2 ± 4.6 ^a^	74.8 ± 4.6 ^a^	ns ^b^	73.9 ± 3.5 ^a^	73.0 ± 3.9 ^a^	ns ^b^
BMI (kg/m^2^)	26.6 ± 1.85 ^a^	28.8 ± 1.8 ^a^	ns ^b^	26.0 ± 1.7 ^a^	26.0 ± 1.7 ^a^	ns ^b^
Resistance (ohm)	493.7 ± 21.3 ^a^	480 ± 12.7 ^a^	ns ^b^	566.7 ± 28.6 ^a^	531.0 ± 21.8 ^a^	ns ^b^
Reactance (ohm)	46.7 ± 2.8 ^a^	44.0 ± 0.5 ^a^	ns ^b^	42.0 ± 2.4 ^a^	50.8 ± 2.0 ^a^	ns ^b^
Phase angle (°)	5.4 ± 0.3 ^a^	5.3 ± 0.2 ^a^	ns ^b^	4.3 ± 0.3 ^a^	4.8 ± 0.2 ^a^	ns ^b^
Hydration status:						
TBW (%)	56.2 ± 1.9 ^a^	56.8 ± 1.6 ^a^	ns ^b^	50.8 ± 1,1 ^a^	48.7 ± 2.0 ^a^	ns ^b^
ICW (%)	51.2 ± 1.8 ^a^	50.7 ± 1.3 ^a^	ns ^b^	45.3 (29.9–51.6) ^c^	47.7 ± 1.3 ^a^	ns ^d^
ECW (%)	48.8 ± 1.8 ^a^	49.3 ± 1.1 ^a^	ns ^b^	54.7 (48.4–55.7) ^c^	52.3 ± 1.3 ^a^	ns ^d^

^a^ Data expressed as mean ± standard deviation; ^b^ Applied test: *t*-test for paired data; ^c^ Data expressed as a median and the minimum-maximum range is shown in brackets; ^d^ Applied test: Wilcoxon test; Values of *p* ≤ 0.05 are considered statistically significant. Abbreviations: BMI, body mass index; TBW, total body water; ICW, intra cell water; ECW, extra cell water.

**Table 6:** Body composition assessment of control group divided into two subgroups according to gender.

		Male Patients			Female Patients	
	T0	T1	T0 vs. T1	T0	T1	T0 vs. T1
Weight (kg)	73.1 ± 3.9 ^a^	73.2 ± 4.3 ^a^	ns ^b^	73.5 ± 3.4 ^a^	73.4 ± 3.0 ^a^	ns ^b^
BMI (kg/m^2^)	26.1 ± 1.9 ^a^	26.8 ± 1.7 ^a^	ns ^b^	25.8 ± 1.8 ^a^	25.6 ± 1.7 ^a^	ns ^b^
Resistance (ohm)	489.7 ± 13.3 ^a^	490 ± 11.6 ^a^	ns ^b^	570.7 ± 27.5 ^a^	531.0 ± 21.8 ^a^	ns ^b^
Reactance (ohm)	45.9 ± 2.6 ^a^	45.3 ± 1.7 ^a^	ns ^b^	43.1 ± 2.5 ^a^	45.4 ± 3.4 ^a^	ns ^b^
Phase angle (°)	5.5 ± 0.2 ^a^	5.4 ± 0.2 ^a^	ns ^b^	4.4 ± 0.4 ^a^	4.5 ± 0.3 ^a^	ns ^b^
Hydration status:						
TBW (%)	56.2 ± 1.9 ^a^	56.8 ± 1.6 ^a^	ns ^b^	50.8 ± 1,1 ^a^	48.7 ± 2.0 ^a^	ns ^b^
ICW (%)	50.6 ± 1.8 ^a^	50.8 ± 1.7 ^a^	ns ^b^	47.2 ± 1.5 ^a^	48.1 ± 1.4 ^a^	ns ^b^
ECW (%)	49.4 ± 1.9 ^a^	49.2 ± 1.3 ^a^	ns ^b^	52.8 ± 1.5 ^a^	51.9 ± 1.3 ^a^	ns ^b^

^a^ Data expressed as mean ± standard deviation; ^b^ Applied test: *t*-test for paired data; Values of *p* ≤ 0.05 are considered statistically significant. Abbreviations: BMI, body mass index; TBW, total body water; ICW, intra cell water; ECW, extra cell water.

**Table 7:** Oxidative stress and antioxidant defense mechanism efficiency assessment of the OFS group.

		Male Patients			Female Patients	
	T0	T1	T0 vs. T1	T0	T1	T0 vs. T1
FORT (U)	261.4 ± 26.3 ^a^	160 (160–250) ^c^	*p* = 0.00391 ^d^	268 ± 46.2 ^a^	312 ± 46.1 ^a^	*p* = 0.0172 ^b^
FORD (mmol/L Trolox)	0.88 ± 0.1 ^a^	1.43 ± 0.03 ^a^	*p* = 0.0030 ^b^	1.29 ± 0.2 ^a^	1.37 ± 0.1 ^a^	ns ^b^

^a^ Data expressed as mean ± standard deviation; ^b^ Applied test: *t*-test for paired data; ^c^ Data expressed as a median and the minimum-maximum range is shown in brackets; ^d^ Applied test: Wilcoxon test; Values of *p* ≤ 0.05 are considered statistically significant. Abbreviations: FORT, Free Oxygen Radical Test; FORD, Free Oxygen Radical Defense; ns, not significant.

**Table 8:** Oxidative stress and antioxidant defense mechanism efficiency assessment of the control group.

		Male Patients			Female Patients	
	T0	T1	T0 vs. T1	T0	T1	T0 vs. T1
FORT (U)	271.5 ± 27.4 ^a^	269.2 ± 30.3 ^a^	ns ^b^	259.1 ± 51.3 ^a^	260.4 ± 40.6 ^a^	ns ^b^
FORD (mmol/L Trolox)	0.71 ± 0.3 ^a^	1.10 ± 0.5 ^a^	ns ^b^	1.34 ± 0.1 ^a^	1.45 ± 0.7 ^a^	ns ^b^

^a^ Data expressed as mean ± standard deviation; ^b^ Applied test: *t*-test for paired data; Values of *p* ≤ 0.05 are considered statistically significant. Abbreviations: FORT, Free Oxygen Radical Test; FORD, Free Oxygen Radical Defense; ns, not significant.

**Table 9:** PREDIMED and IPAQ questionnaires of study population.

	PREDIMED		
	T0	T1	*p* (McNemar’s Test)
Minimal adherence (%)	0	0	ns
Average adherence (%)	52.2	56.5	ns
Maximal adherence (%)	47.8	43.5	ns
	**IPAQ**		
	**T0**	**T1**	***p* (McNemar’s Test)**
Inactive (%)	65.2	60.8	ns
Sufficiently active (%)	34.8	39.2	ns
Very active (%)	0	0	ns

Abbreviation: ns, not significant.

## Data Availability

Data available on request due to privacy restrictions. The data presented in this study are available on request from the corresponding author.
